# Transcriptome Analysis of Green and White Leaf Ornamental Kale Reveals Coloration-Related Genes and Pathways

**DOI:** 10.3389/fpls.2022.769121

**Published:** 2022-04-27

**Authors:** Fuhui Zhou, Yang Liu, Xin Feng, Yuting Zhang, Pengfang Zhu

**Affiliations:** ^1^College of Forestry, Shenyang Agricultural University, Shenyang, China; ^2^Key Laboratory of Forest Tree Genetics, Breeding and Cultivation of Liaoning Province, Shenyang, China

**Keywords:** transcriptome, chlorophyll, carotenoid, chloroplast development, photosynthesis

## Abstract

Leaf color is a crucial agronomic trait in ornamental kale. However, the molecular mechanism regulating leaf pigmentation patterns in green and white ornamental kale is not completely understood. To address this, we performed transcriptome and pigment content analyses of green and white kale leaf tissues. A total of 5,404 and 3,605 different expressed genes (DEGs) were identified in the green vs. white leaf and the green margin vs. white center samples. Kyoto Encyclopedia of Genes and Genome (KEGG) pathway enrichment analysis showed that 24 and 15 common DEGs in two pairwise comparisons were involved in chlorophyll metabolism and carotenoid biosynthesis, respectively. Seventeen genes related to chlorophyll biosynthesis were significantly upregulated in green leaf tissue, especially *chlH* and *por*. Of the 15 carotenoid biosynthesis genes, all except *CYP707A* and *BG1* were lower expressed in white leaf tissue. Green leaf tissue exhibited higher levels of chlorophyll and carotenoids than white leaf tissue. In addition, the DEGs involved in photosystem and chlorophyll-binding proteins had higher expression in green leaf tissue. The *PSBQ*, *LHCB1.3*, *LHCB2.4*, and *HSP70* may be key genes of photosynthesis and chloroplast formation. These results demonstrated that green and white coloration in ornamental kale leaves was caused by the combined effects of chlorophyll and carotenoid biosynthesis, chloroplast development, as well as photosynthesis. These findings enhance our understanding of the molecular mechanisms underlying leaf color development in ornamental kale.

## Introduction

Ornamental kale (*Brassica oleracea* L. var. *acephala*), also known as “leaf peony,” is a variety of *B*. *oleracea* (Liu et al., 2020). In recent years, this ornamental plant has gained wide popularity as an attractive decorative plant and used in potted or cut form due to its colorful leaves of various shapes and having strong resistance to cold (Feng et al., 2020). Usually, the rosette period is the best viewing period, as the ornamental kale leaves grow vigorously during this period. Ornamental kale is also edible, and it contains many bioactive compounds such as glucosinolates, phenolic compounds, and carotenoids. These compounds have long been considered to have strong antioxidant capacities and are beneficial to human health (Jeon et al., 2018; Jin et al., 2018; Guo et al., 2019; Liu et al., 2020). One of the most important agronomic characteristics of ornamental kale is the color of its leaves, which can be green, white, pink, purple, or other colors, with the colors sometimes arranged in complex patterns (Zhang et al., 2008; Ren et al., 2015; Feng et al., 2021). Some ornamental kale varieties exhibit changes in leaf color patterns during different developmental stages and under different environmental conditions (Jin et al., 2018). Plant pigments play an important role in plant coloration. The major classes of these pigments include chlorophylls, carotenoids, anthocyanins, and betalains. Of these, chlorophylls and carotenoids also play essential roles in photosynthesis (Simon, 1997).

In recent years, an increasing number of studies have focused on leaf color in ornamental kale. Zhu et al. (2016) localized the pink-leaf gene (*Pi*) to the top of chromosome C3. Liu et al. (2017) performed fine mapping of the purple leaf gene *BoPr*, and Zhang et al. (2012) found that *BoPAP1* is important for anthocyanin accumulation in purple kale. Jeon et al. (2018) demonstrated that red kale contains more anthocyanins and phenylpropanoids than green kale, whereas green kale has higher carotenoid content. However, most of these studies have focused on the mechanisms underlying the formation of pink, purple, and red leaf coloration, only a few studies on the white leaf. The white ornamental kale is a temperature-sensitive chlorophyll mutant. The color of the cultivar will turn completely white when under low-temperature conditions, and can turn green again under normal temperature conditions (>16°C). Recent studies have shown that the white phenotype might be caused by inhibiting chlorophyll biosynthesis and chloroplast development (Zhou et al., 2013; Yan et al., 2020). However, the study on the color development detailed molecule mechanism of white and green leaf ornamental kale has not been reported.

In the current study, we explored the molecular mechanism underlying white and green leaf color formation in ornamental kale. To investigate this mechanism in detail, we performed the transcriptome profiling to compare the transcript profile of ‘G02’ with that of ‘1631’, and the transcript profile of the green margin with that of the white center in ‘D11’ leaves. We also measured the contents of major pigments in the leaves and identified key different expressed genes (DEGs) in green vs. white leaf tissue. Our findings provide important transcriptomic information to help uncover the molecular mechanism regulating green and white leaf coloration in ornamental kale.

## Materials and Methods

### Plant Material

**FIGURE 1:**
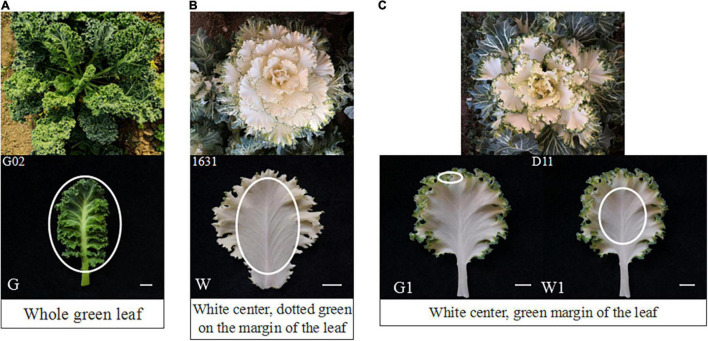
Phenotypes and sampling sites of the three ornamental kale cultivars investigated. **(A)** Phenotype of ‘G02’. **(B)** Phenotype of ‘1631’. **(C)** Phenotype of ‘D11’. The white ovals indicate the sampling sites: G, ‘G02’ plant; W, white center of ‘1631’ plant; G1, green margin of ‘D11’ plant; W, white center of ‘D11’ plant. Scale bar represents 1 cm.

The green ornamental kale cultivar ‘G02’, white cultivar ‘1631’, and white-green bicolor cultivar ‘D11’ (green margin and white center) used in this study were obtained from the germplasm nursery of Shenyang Agricultural University and grown in a greenhouse located in Shenyang, China. The materials were planted in August 2019 and harvested at the rosette stage (December 2019). During this period, the color of ornamental kale is stable and it is the best viewing period. Sample of leaves without the main vein was collected, including whole ‘G02’ leaves, the white center of ‘1631’ leaves, the green margin of ‘D11’ leaves, and the white center of ‘D11’ leaves (Figure 1). After measurement of the chlorophyll and carotenoid contents, the samples were immediately frozen in liquid nitrogen and stored at −80°C for later RNA isolation. Three independent biological replicates per sample were used for analysis.

### RNA Extraction, cDNA Library Construction, and Sequencing

Total RNA was extracted from mixed leaf tissue samples from four individual plants using RNAiso reagent (TaKaRa Shuzo Co. Ltd., Japan) according to the manufacturer’s instructions. The integrity and purity of the RNA were examined by agarose gel electrophoresis and measured with a NanoDrop 8000 spectrophotometer (Thermo Scientific, United States).

High-quality RNA from each sample was used for library construction following the manufacturer’s protocol (Illumina, United States). Briefly, oligo (dT) magnetic beads were used to obtain purified poly-A mRNA, which was fragmented into small pieces in fragmentation buffer. These small fragments were used as templates for first-strand cDNA synthesis with random hexamer primers. Second-strand cDNA was synthesized using DNA polymerase I, RNase H, dNTP, and buffer. Short cDNA fragments were purified with a QiaQuick PCR extraction kit. Following end repair and the addition of poly(A), the cDNA fragments were attached to adapters (Illumina). After selecting suitable ligated cDNA fragments, PCR amplification was performed and the PCR products were purified. The cDNA libraries were sequenced on the Illumina HiSeq™ 2000 platform.

### Analysis of RNA Sequencing Data

To obtain high-quality clean reads, the adaptor sequences, unknown bases (>10% N bases), repeats, and low-quality reads (>50% bases with quality value ≤5) were removed from the raw reads obtained by RNA sequencing (RNA-seq). The reads were aligned to the *B. oleracea* reference genome^1^ using HISAT software (Kim et al., 2015).

Different expressed genes were identified using DESeq. *Q*-value < 0.01 and | log_2_(fold change)| > 1 were selected as thresholds determining the significance of differential expression. Gene Ontology (GO) and Kyoto Encyclopedia of Genes and Genome (KEGG) were used for functional annotation, classification, and pathway enrichment analysis of the DEGs.

### Quantitative Real-Time PCR

To validate the reliability of transcriptome sequencing results, 12 DEGs (log_2_FoldChange ≥ 2 in G vs. W or G1 vs. W1) were selected for quantitative real-time PCR (qRT-PCR) validation. Among them, were eight porphyrin and chlorophyll metabolism genes (Figures 7A–H), and four carotenoid biosynthesis genes (Figures 7I–L).

**FIGURE 2:**
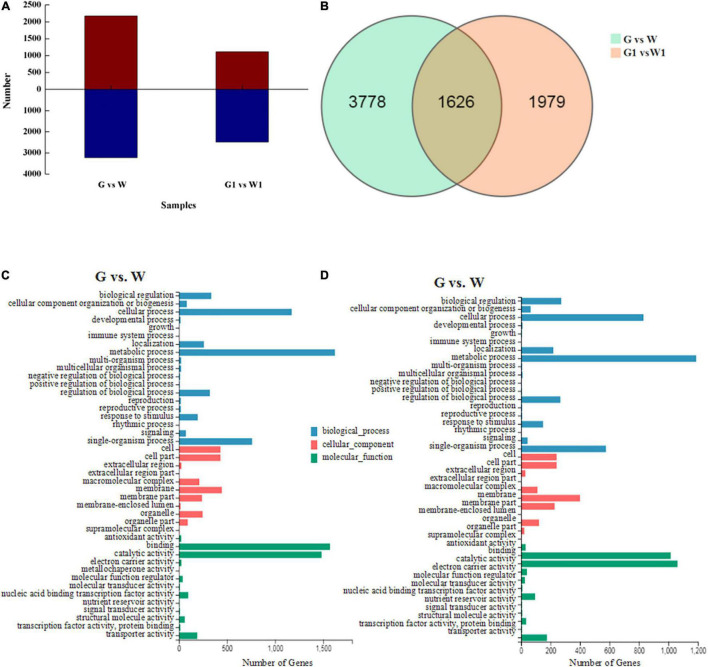
**(A)** Numbers of up- and downregulated DEGs identified in pairwise comparisons. Red, upregulated DEG; blue, downregulated DEGs. **(B)** Venn diagram of DEGs identified by pairwise comparisons. GO classifications of DEGs in the **(C)** G vs. W and **(D)** G1 vs. W1 comparisons.

**FIGURE 3:**
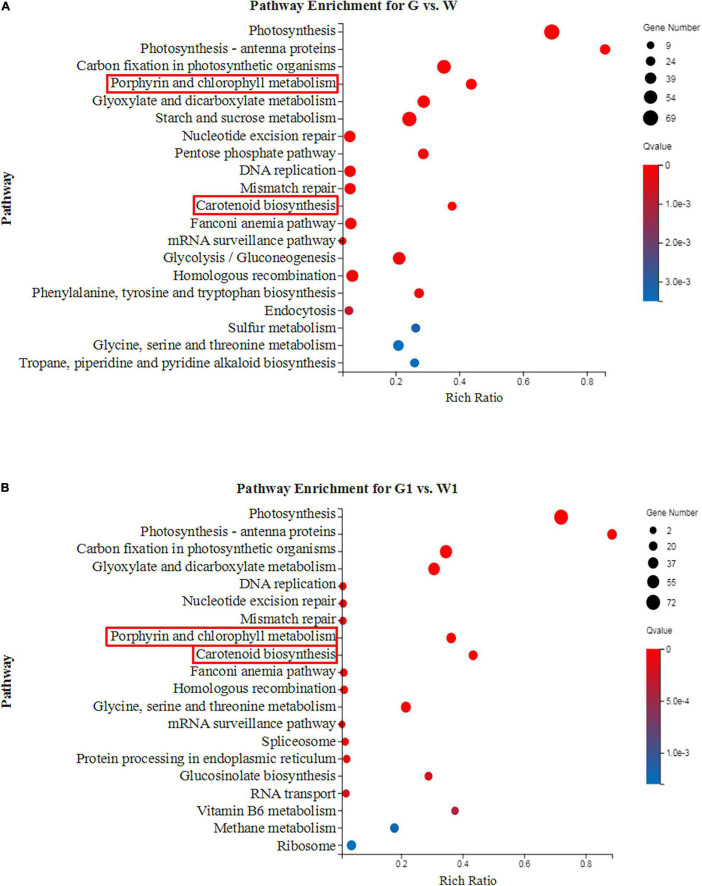
The top 20 enriched KEGG pathways of the DEGs between green and white kale leaf tissues. **(A)** G vs. W and **(B)** G1 vs. W1.

**FIGURE 4:**
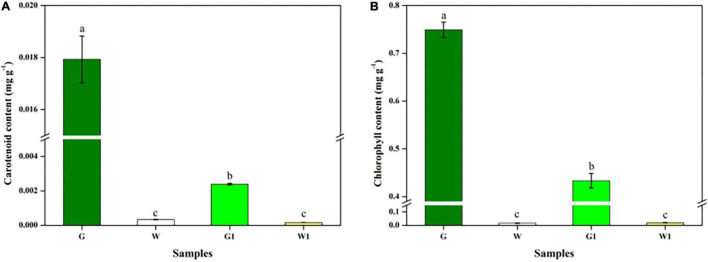
Chlorophyll and carotenoid differed significantly between green and white kale leaf tissues. **(A)** Chlorophyll contents. **(B)** Carotenoid contents. Vertical bars represent the standard deviation of the means (*n* = 3). Different letters: statistically significant differences (*P* < 0.05).

**FIGURE 5:**
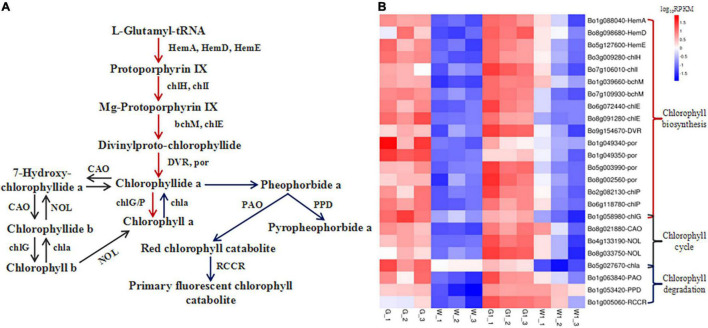
Genes encoding enzymes involved in chlorophyll metabolism are differentially expressed in green vs. white kale leaf tissues. **(A)** Diagrammatic representation of the chlorophyll metabolism pathway involving the DEGs in G vs. W and G1 vs. W1. **(B)** Heatmap of the expression patterns of DEGs involved in chlorophyll metabolism in the four samples. Red and blue tiles indicate higher and lower FPKM values, respectively.

**FIGURE 6:**
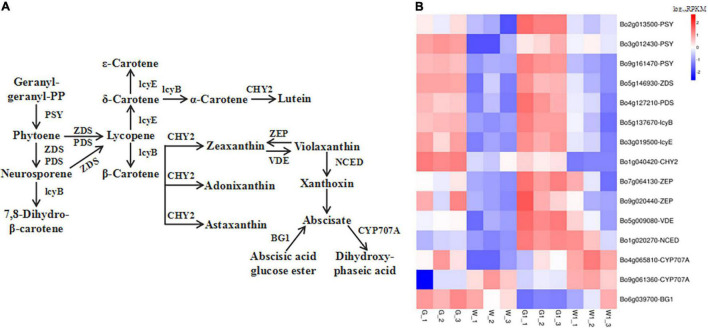
Genes encoding enzymes involved in carotenoid metabolism are differentially expressed in green vs. white kale leaf tissues. **(A)** Diagrammatic representation of the carotenoid biosynthesis pathway involving the DEGs in G vs. W and G1 vs. W1. **(B)** Heatmap of the expression patterns of DEGs involved in carotenoid biosynthesis in the four samples. Red and blue tiles indicate higher or lower FPKM values, respectively.

**FIGURE 7:**
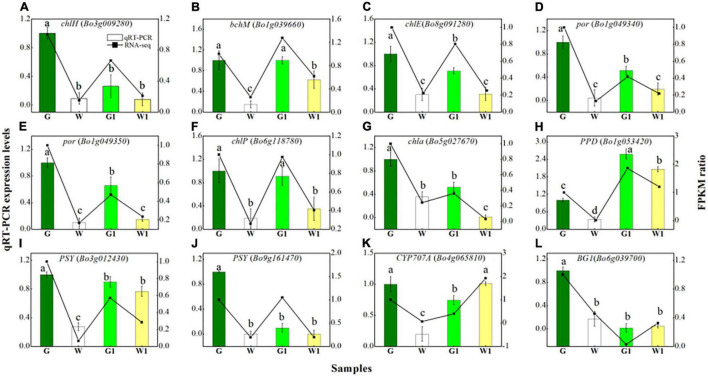
Quantitative real-time PCR validation of eight DEGs **(A–H)** related to porphyrin and chlorophyll metabolism and four DEGs **(I–L)** related to carotenoid biosynthesis in four sample groups. Vertical bars represent the standard deviation of the means (*n* = 3). Different letters: statistically significant differences (*P* < 0.05).

The gene-specific primer sequences are shown in Supplementary Table 1. AMV First Strand cDNA Synthesis Kit (Shanghai Sangon Biotechnology Co., Ltd.) was used for the first-strand cDNA synthesis. The 21 μL of reaction mixture included total RNA (6 μL), Oligo dT primer (1 μL), RNase-free water (5 μL), five Reaction buffer (4 μL), Rnase Inhibitor (1 μL), dNTP Mix (2 μL), and AMV RT (2 μL). qRT-PCR reactions were performed with Roche LightCycler 480 (Roche, Bazel, Switzerland) thermocycle under the following conditions: 95°C for 30 s, 40 cycles of 95°C for 5 s, and 60°C for 30 s. The reactions comprised of 10 μL of 2TB Green Premix Ex Taq II (Takara, Dalian, China), 2 μL of cDNA template, 1 μL of forward/reverse primers, 0.4 μL of ROX Reference Dye, and 5.6 μL of RNase-free water. The gene relative expression level of the selected gene was analyzed by the 2^–ΔΔCT^ method (Livak and Schmittgen, 2001). Each experiment was conducted with three technical replicates.

### Measuring Chlorophyll and Carotenoid Contents

The chlorophyll and carotenoid contents of the samples were measured as described by Zhu et al. (2017), with minor modifications. Fresh samples (0.5 g) were ground with 3 mL of 95% ethanol solution and a little calcium carbonate and quartz sand in a mortar. After rapid grinding, the mixture was filtered and the mortar was continuously rinsed. Finally, added 95% ethanol solution to the supernatant to 15 mL. Pigment contents were measured in a spectrophotometer based on absorbance (*A*) at 470, 649, and 665 nm, using the following equations:


$$\mathrm{Chlorophyll}\,\mathrm{content}\,\left(\mathrm{mg}\,g^{-1}\,\text{FW}\right)=\left({\text{C}}_{\text{chl}}\times V\times n\right)/m$$

$${\text{C}}_{\text{chl}}={\text{C}}_{\text{a}}+{\text{C}}_{\text{b}}$$

$$\mathrm{Chlorophyll}\,a\,\mathrm{content}\,\left(\mathrm{mg}\,g^{-1}\,\text{FW}\right)=\;\,13.95\,A_{665\,\text{nm}}-6.88\,A_{649\,\text{nm}}$$

$$\text{Chlorophyll}\,b\,\mathrm{content}\,\left(\mathrm{mg}\,g^{-1}\,\text{FW}\right)=\;\,24.96\,A_{649\,\text{nm}}-7.32\,A_{665\,\text{nm}}$$

$$\text{Carotenoid}\,\text{content}\,\left(\mathrm{mg}\,g^{-1}\,\text{FW}\right)=\left(1000\,A_{470}-2.05\,C_{\text{a}}-114.8\,C_{\text{b}}\right)\times V\times n/245\,m$$

where C_a_ is chlorophyll a, C_b_ is chlorophyll b, *V* is the total volume of extract (mL), *n* is the dilution multiple, and *m* is the sample weight (g). Three technical replicates were conducted for each sample.

### Statistical Analyses

The data for pigment contents and gene relative expression levels represent the mean ± standard deviation (SD). SPSS software (version 22.0, SPSS Inc., United States) was used to perform an LSD test to compare the pigment contents and gene relative expression levels of the four groups of samples. Differences were considered to be statistically significant at *P* < 0.05.

## Results

### RNA Sequencing and Different Expressed Gene Analyses

To gain a global view of the transcriptome profiles of ornamental kale in different colors, 12 cDNA libraries from three biological replicates of ‘G02’ (G), ‘1631’ (W), the green margins of ‘D11’ leaves (G1), and the white centers of ‘D11’ leaves (W1) were constructed. We obtained 544.90 million raw reads. After filtering, 532.62 million high-quality clean reads were obtained (Table 1).

**TABLE 1:** Summary of sequencing and assembly statistics for the 12 transcriptome data from different ornamental kale samples.

Samples ID	Total raw reads (×10^6^)	Total clean reads (×10^6^)	Total clean bases (Gb)	Clean reads Q30 (%)	Total mapping (%)	Uniquely mapping (%)
G_1	50.16	48.98	7.27	94.45	90.22	87.34
G_2	46.53	45.32	6.71	94.53	89.71	85.72
G_3	51.56	50.24	7.39	94.86	90.12	87.09
G1_1	40.36	39.6	5.87	94.74	91	87.02
G1_2	43.94	43.06	6.34	94.85	90.38	86.04
G1_3	48.71	47.53	7.00	94.68	89.86	86.08
W_1	44.90	43.96	6.46	94.85	90.12	87.06
W_2	44.63	43.79	6.49	94.74	89.77	86.18
W_3	43.41	42.24	6.21	94.54	89.23	85.64
W1_1	44.67	43.44	6.43	94.45	89.64	86.16
W1_2	43.22	42.32	6.28	94.38	89.71	85.97
W1_3	42.81	42.14	6.26	94.72	90.09	86.84
Total	544.90	532.62				

We selected DEGs from two comparison groups (G vs. W and G1 vs. W1) based on FPKM values. A total of 5,404 DEGs (2,170 upregulated and 3,234 downregulated genes) and 3,605 DEGs (1,115 upregulated and 2,490 downregulated genes) were identified in G vs. W and G1 vs. W1, respectively (Figure 2A). Among these, 1,626 DEGs were common to both comparison groups, 3,778 were specific to G vs. W, and 1,979 were specific to G1 vs. W1 (Figure 2B). We performed GO analysis to classify the DEGs into three GO categories: biological process (BP), cellular component (CC), and molecular function (MF). The DEGs in both comparisons were assigned to similar GO terms (Figures 2C,D). The most highly enriched term in the BP category was “metabolic process,” followed by “cellular process” and “single-organism process.” In the CC category, the most highly enriched term was “membrane.” Among the MF category, the most highly enriched GO term was “binding,” followed by “catalytic activity.”

To further analyze the functional differences in the DEGs identified by pairwise comparisons, we performed KEGG enrichment analysis. The DEGs in G vs. W and G1 vs. W1 were enriched in 277 and 263 KEGG pathways, respectively. The top 20 most highly enriched pathways were shown in Figure 3. Both “Porphyrin and chlorophyll metabolism” and “Carotenoid biosynthesis” related to pigments and were significantly enriched in the two comparison groups. Thus we investigated these two pathways in our subsequent analysis.

### Chlorophyll and Carotenoid Contents

The G, W, G1, and W1 groups exhibited distinct chlorophyll and carotenoid contents (Figures 4A,B). These two pigments were much more abundant in green leaf (G and G1) vs. white leaf tissue (W and W1); the G group had notably higher chlorophyll and carotenoid levels than the three other groups (*P* < 0.05). There was no significant difference in chlorophyll and carotenoid content between two white leaf tissue samples (*P* > 0.05).

### Analysis of Genes Involved in Porphyrin and Chlorophyll Metabolism

We identified 35 and 29 DEGs involved in porphyrin and chlorophyll metabolism in G vs. W and G1 vs. W1, respectively. Of these, 24 common DEGs are involved in chlorophyll biosynthesis, the chlorophyll cycle, and chlorophyll degradation (Supplementary Table 2 and Figure 5). DEGs involved in the chlorophyll biosynthesis pathway encode HemA (glutamyl-tRNA reductase), HemD (uroporphyrinogen-III synthase), HemE (uroporphyrinogen decarboxylase), chlH (magnesium chelatase subunit H), chlI (magnesium chelatase subunit I), bchM (magnesium-protoporphyrin O-methyltransferase), chlE [magnesium-protoporphyrin IX monomethyl ester (oxidative) cyclase], DVR (divinyl chlorophyllide a 8-vinyl-reductase), por (protochlorophyllide reductase), chlG (chlorophyll/bacteriochlorophyll a synthase), and chlP (geranylgeranyl diphosphate/geranylgeranyl-bacteriochlorophyllide a reductase). DEGs involved in the chlorophyll cycle encode chlG, CAO (chlorophyllide a oxygenase), NOL [chlorophyll(ide) b reductase], and chla (chlorophyllase). Finally, DEGs involved in chlorophyll degradation encode chla, PAO (pheophorbide a oxygenase), PPD (pheophorbidase), and RCCR (red chlorophyll catabolite reductase). All these DEGs were expressed at significantly higher levels in G and G1 than that in W and W1. In the G group, the gene *PAO* was expressed at the lowest level, followed by *HemD*, *RCCR*, and *PPD*. The gene *PAO* was expressed at the lowest level in the G1 group. The genes *chla* and *chlP* (*Bo2g082130*) were expressed at the highest levels in W and W1, respectively. The gene *BCM1* (*BALANCE of CHLOROPHYLL METABOLISM*, *Bo3g028240* and *Bo4g038770*) involved in chlorophyll degradation also showed higher expression in green leaf (Table 2). No significant difference was found in the expression of the *STAY-GREEN* (SGR) *gene* (*Bo3g045460*) in two pairwise comparisons. Based on the above results, we concluded that the green leaf tissue had a higher chlorophyll metabolism level, and chlorophyll biosynthesis played a critical role.

**TABLE 2:** Genes encoding photosystem proteins, chlorophyll binding proteins and others.

Unigene ID	Log_2_ (W/G)	Log_2_ (W1/G1)	Up-down	Description
**Photosystem proteins**				
*Bo5g019300*	–9.691	–3.328	Down	PSBQ (Photosystem II subunit Q)
*Bo1g158910*	–7.195	–6.36	Down	PSBQ (Photosystem II subunit Q)
*Bo5g155140*	–6.647	–5.832	Down	PSBQ (Photosystem II subunit Q)
*Bo8g021860*	–4.458	–4.553	Down	PSBS (Photosystem II subunit S)
*Bo6g034780*	–4.307	–3.226	Down	PSAG (Photosystem I subunit G)
*Bo6g003410*	–3.5	–3.406	Down	PSBS (Photosystem II subunit S)
*Bo1g024020*	–3.491	–3.444	Down	PSBQ (Photosystem II subunit Q)
*Bo9g017950*	–3.449	–2.649	Down	PSAN (Photosystem I subunit N)
*Bo5g018220*	–3.431	–3.589	Down	PSBQ-Like (Photosystem II subunit Q)
*Bo3g148990*	–3.289	–3.855	Down	PSAK (Photosystem I subunit K)
*Bo6g105200*	–3.053	–2.292	Down	PSBY (Photosystem II subunit Y)
*Bo8g114480*	–2.976	–2.436	Down	PSBP1 (Photosystem II subunit P1)
*Bo6g098700*	–2.92	–3.412	Down	PSBY (Photosystem II subunit Y)
*Bo1g017150*	–2.861	–2.092	Down	PSAE1 (Photosystem I subunit E1)
*Bo4g134490*	–2.749	–2.454	Down	PSAL (Photosystem I subunit L)
*Bo3g102750*	–2.625	–2.002	Down	PSAN (Photosystem I subunit N)
*Bo5g007740*	–2.617	–2.89	Down	PSBW (Photosystem II subunit W)
*Bo8g034270*	–2.581	–2.642	Down	PSAL (Photosystem I subunit L)
*Bo9g076240*	–2.547	–2.023	Down	PSBQ2 (Photosystem II subunit Q2)
*Bo2g056130*	–2.541	–2.541	Down	PSBY (Photosystem II subunit Y)
*Bo8g007190*	–2.521	–2.3	Down	PSBP1 (Photosystem II subunit P1)
*Bo9g021880*	–2.422	–2.233	Down	PSBO1 (Photosystem II subunit O1)
*Bo6g122520*	–2.376	–3.08	Down	PSBR (Photosystem II subunit R)
*Bo8g011060*	–2.315	–2.549	Down	PSAO (Photosystem I subunit O)
*Bo8g112500*	–2.291	–2.049	Down	PSAO (Photosystem I subunit O)
*Bo6g051400*	–2.291	–2.267	Down	PSAG (Photosystem I subunit G)
*Bo5g062900*	–2.283	–2.343	Down	PSAK (Photosystem I subunit K)
*Bo8g054500*	–2.269	–2.34	Down	PSAE1 (Photosystem I subunit E1)
*Bo3g181110*	–2.161	–2.259	Down	PSAH2 (Photosystem I subunit H2)
*Bo3g185460*	–2.002	–2.408	Down	PSAG (Photosystem I subunit G)
*Bo2g167690*	–2.001	–2.014	Down	PSBO1 (Photosystem II subunit O1)
**Chlorophyll binding proteins** (LHC: light-harvesting chlorophyll)				
*Bo7g044900*	–11.955	–3.61	Down	LHCB1.3
*Bo7g085890*	–5.681	–3.508	Down	LHCB2.4
*Bo2g110390*	–5.466	–2.069	Down	LHCB5
*Bo9g008350*	–5.453	–3.424	Down	LHCB2.4
*Bo2g144900*	–5.435	–3.869	Down	LHCB2.4
*Bo4g039810*	–4.75	–2.866	Down	LHCB1.4
*Bo8g096010*	–4.37	–2.678	Down	LHCA2
*Bo4g101180*	–4.217	–2.283	Down	LHCA2
*Bo4g086780*	–4.034	–2.258	Down	LHCA3
*Bo3g085600*	–4.034	–2.4	Down	LHCB2.2
*Bo3g027740*	–3.84	–2.728	Down	LHCB1.4
*Bo4g039820*	–3.758	–2.651	Down	LHCB1.4
*Bo3g146480*	–3.733	–2.266	Down	LHCB1.3
*Bo8g105860*	–3.731	–2.459	Down	LHCB6
*Bo5g061110*	–3.261	–2.069	Down	LHCB1.1
*Bo3g134510*	–3.205	–2.311	Down	LHCA4
*Bo5g139650*	–2.963	–2.101	Down	LHCB4.2
*Bo3g044670*	–2.948	–2.063	Down	LHCB5
*Bo2g001220*	–2.893	–2.244	Down	LHCB4.1
*Bo8g068320*	–2.84	–2.807	Down	LHCA6
*Bo6g068340*	–2.814	–2.232	Down	LHCA1
*Bo9g088290*	–2.533	–2.005	Down	LHCB5
*Bo8g087140*	–2.53	–2.448	Down	LHCA1
*Bo9g042070*	–2.365	–2.246	Down	LHCA3
*Bo4g027600*	–2.074	–2.198	Down	LHCB5
*Bo4g025550*	–2.001	–2.588	Down	LHCB4.3
**Others**				
*Bo1g037850*	13.859	3.155	Up	HSP70-7 (heat shock protein 70)
*Bo1g139130*	5.019	2.087	Up	HSP70 (heat shock protein 70)
*Bo4g038770*	–2.576	–2.175	Down	BCM1 (BALANCE of CHLOROPHYLL METABOLISM1)
*Bo3g028240*	–2.109	–1.576	Down	BCM1 (BALANCE of CHLOROPHYLL METABOLISM1)
*Bo7g003410*	–2.472	–1.871	Down	GLK1 (GOLDEN2-LIKE1)
*Bo2g122230*	–2.118	–1.481	Down	GLK2 (GOLDEN2-LIKE2)
*Bo7g067200*	–1.455	/	Down	GLK2 (GOLDEN2-LIKE2)

### Analysis of the Genes Involved in Carotenoid and Abscisic Acid Biosynthesis

We identified 20 and 23 genes related to carotenoid biosynthesis that were significantly differentially expressed in G vs. W and G1 vs. W1, respectively (Supplementary Table 3). Fifteen common DEGs related to carotenoid biosynthesis in two pairwise comparisons were screened (Figure 6). Most were expressed at higher levels in G and G1 vs. the white leaf tissue groups, except for genes *CYP707A* [(+)-abscisic acid 8′-hydroxylase] (*Bo4g065810*, *Bo9g061360*) and *BG1* (β-D-glucopyranosyl abscisate β-glucosidase). Notably, *BG1* was the most highly expressed level in G, W, and W1, and *ZEP* (encoding zeaxanthin epoxidase) (*Bo7g064130*) was the most highly expressed in G1. These results could suggest that green leaf tissue had more active carotenoid biosynthesis than white leaf tissue.

### Expression Analysis of Different Expressed Genes

As shown in Figure 7, the tested genes that participate in the porphyrin and chlorophyll metabolism demonstrated low expression levels in white leaf tissue samples (W and W1), compared to green leaf tissue samples (G and G1). Genes of *PSY* (*Bo3g012430* and *Bo9g161470*) had higher expression levels in the G and G1 than that in the W and W1, but *CYP707A* (*Bo4g065810*) and *BG1* had higher expression levels in the W1 than that in the G1. The expression patterns of 12 genes measured *via* qRT-PCR were similar to the RNA-seq results, although relative expression levels displayed a slightly inconsistent. Overall, consistent regulation patterns were found in qRT-PCR and RNA-seq indicated the data was reliable.

### Analysis of Genes Involved in Chloroplast Formation

To further explore potential candidate genes involved in leaf color formation, DEGs [|log_2_(fold change)|>2] encoding photosystem proteins, chlorophyll binding proteins and others for two comparison groups are listed in Table 2. Genes involved in photosystem and chlorophyll binding proteins were downregulated expression in white leaf tissue samples (W and W1); however, *HSP70* were upregulated. In photosystem proteins, 14 genes were involved in encoding photosystem I proteins and 17 genes were involved in encoding photosystem II proteins. It is worth noting that six genes encoding PSBQ (Photosystem II subunit Q) had significantly different expression levels in both comparison groups. Genes annotated as *GOLDEN2-LIKE* (*GLK*) (*Bo7g00341*0, *Bo2g122230*, and *Bo7g067200*) were downregulated expression in white leaf tissue samples (W and W1) (Table 2). The lower expression level of these genes may inhibit the chloroplast development in the white leaf, and the HSP70 was a negative regulator of chloroplast formation.

## Discussion

Ornamental kale is both an attractive landscape plant and a nutritious vegetable. Because its beautiful colors remain vibrant at 15–20°F, ornamental kale has a long-lasting ornamental period (Zhu et al., 2016; Jin et al., 2018). In general, leaf coloration in kale is mainly due to the accumulation of chlorophylls, carotenoids, and anthocyanins. Zhou et al. (2013) have found that the inhibition of chloroplast development and chlorophyll biosynthesis results in the formation of the white leaf. However, little is known about the different patterns of gene expression of green vs. white ornamental kale leaves. In the current study, we performed transcriptome analysis to explore the molecular mechanism underlying green and white leaf color variation. To obtain a comprehensive dataset, we selected a green cultivar (G02) and a white cultivar (1631), as well as the green margin and white center of ‘D11’ leaves, as two comparison groups to identify differences in common gene modules. After confirming the reliability of the data, we conducted further analyses.

We identified 5,404 and 3,605 DEGs in G vs. W and G1 vs. W1, respectively. Fewer DEGs were identified in the G1 vs. W1 comparison, perhaps because the G1 and W1 samples were taken from the same plant. Interestingly, most DEGs were downregulated in G and G1 and upregulated in W and W1. A total of 1,621 and 1,188 DEGs were grouped into the metabolic process category in the two comparison groups, implying that metabolic processes play vital roles in regulating leaf pigmentation-unsurprisingly, given that leaf color is generally associated with pigment metabolism. In the present study, we identified two pathways related to color variations that were enriched in both comparison groups: the “porphyrin and chlorophyll metabolism” and “carotenoid biosynthesis” pathways. We examined the expression patterns of common DEGs in these two pathways in more detail to explore the molecular mechanism underlying pigmentation formation in green and white leaves.

Chlorophyll, a crucial photosynthetic pigment, is responsible for green pigmentation in ornamental kale (Ren et al., 2019; Liu et al., 2020). Much is known about chlorophyll metabolism in plants, including chlorophyll biosynthesis, the chlorophyll cycle, and the chlorophyll degradation pathway (Eckhardt et al., 2004). We identified 24 common DEGs involved in porphyrin and chlorophyll metabolism in the two comparison groups, all of which were upregulated in green (G and G1) vs. white samples. These results suggested that chlorophyll metabolism occurred at higher levels in green leaf tissue, which may contribute to its higher chlorophyll content relative to white leaf tissue.

Among these 24 DEGs, 17 are involved in chlorophyll biosynthesis, 5 in the chlorophyll cycle, and 4 in chlorophyll degradation. *HemA*, *HemD*, and *HemE* are upstream genes of chlorophyll biosynthesis. Previous reports have found in *HemA* mutants, chlorophyll level was reduced (Janina et al., 2014). Mutations in *chlH*, *chlD*, and *chlI* cause disruptions in chlorophyll biosynthesis. *chlH* mutation is common in many chlorophyll-deficient mutants such as the golden leaf Chinese cabbage and yellow-leaf *Brassica napus* (Fu et al., 2019; Zhao et al., 2020). bchM and chlE play major roles in divinylproto-chlorophyllide biosynthesis (Gibson and Hunter, 1994; Tottey et al., 2003). The downstream chlorophyll biosynthesis enzymes por and chlG/P catalyze chlorophyll a biosynthesis (Armstrong et al., 1995; Tanaka et al., 1999; Shalygo et al., 2009). Earlier results have found that por may be a key regulator of chlorophyll biosynthesis in Chinese cabbage (Xie et al., 2018). In this study, all 17 genes were low expressed in the white leaf tissue, and *chlH* and *por* showed obvious different expressions in both comparison groups. These findings suggested that chlorophyll biosynthesis was blocked in the white leaf.

*CAO* is involved in chlorophyll cycle. In the Chinese cabbage mutant (*hy*) with yellow-green leaves, the mutation of *CAO* caused a decrease in chlorophyll content (Liu et al., 2018). Generally, loss of green color in leaves is also the result of chlorophyll degradation (Park et al., 2007), chla, PAO, PPD, and RCCR are key enzymes of chlorophyll degradation (Matile et al., 1999). *BCM* plays a conserved role in attenuating chlorophyll degradation (Wang et al., 2020). In this study, these genes were more highly expressed in green tissue than in white tissue. These results suggested that the lower chlorophyll levels in white leaf tissue were due to the lower expression levels of chlorophyll biosynthesis genes (both upstream and downstream) and the chlorophyll degradation gene did not be a decisive role. These findings were consistent with the observation that the inhibition of chlorophyll biosynthesis was the main reason for the change in leaf color from green to white in ornamental kale (Zhou et al., 2013; Liu et al., 2020) and cauliflower mutant (Zhou et al., 2011).

Carotenoids are naturally occurring pigments found in plants, algae, fungi, and bacteria. These compounds can be synthesized in nearly every type of plastid and are responsible for red, orange, and yellow coloration in plants (Cazzonelli and Pogson, 2010; Zhang et al., 2014). Carotenoids play a variety of key roles in photosynthesis, including harvesting light, protecting chlorophyll from photooxidation, and acting as photoprotectors to help plants adapt to high-light stress (Young, 1991). In the present study, we identified 15 common DEGs involved in carotenoid biosynthesis *via* pairwise comparisons. Most of these DEGs were upregulated in green samples compared to white samples, which likely contributes to the higher carotenoid contents in green vs. white leaf tissue. Moreover, our data displayed that the decreasing trend of carotenoid and chlorophyll content was consistent, which indicates that the decline in content of carotenoids may lead to photooxidative damage to chlorophyll. These observations were consistent with the Chinese kale yellow mutant (Sun et al., 2020).

Carotenoids play essential roles in the biosynthesis of abscisic acid (ABA; Li et al., 2003), a phytohormone that plays a critical role in plant adaptation to various abiotic stresses. ABA has been shown to regulate chlorophyll degradation (Yang et al., 2020a). CYP707A is an ABA 8′-hydroxylase that degrades ABA (Okamoto et al., 2006). BG1 is a β-glucosidase that catalyzes the production of active ABA (Lee et al., 2006). In this study, *CYP707A* and *BG1* presented different expression trends in carotenoid biosynthesis. These results suggested that ABA may play a role in leaf color formation, and genes *CYP707A* and *BG1* represent good candidate genes for involvement in ABA metabolism in ornamental kale. In recent years, only a few studies have investigated the regulating effect of ABA in the color formation of ornamental kale. Ren et al. (2019) found that the red leaves contained higher ABA content than that in green leaves. However, the role of ABA in green and white leaf formation is still poorly understood.

Chloroplast is regarded as the site for photosynthesis and chlorophyll biosynthesis (Yang et al., 2020b). Abnormal chloroplast development is a major reason for the formation of chlorophyll-deficient mutants (Zhang et al., 2020). Previous studies have shown that the white leaf of ornamental kale formation was mainly due to chloroplast deficiency (Zhou et al., 2013; Yan et al., 2020). Chloroplast biogenesis involved several functional processes including thylakoid formation, pigment synthesis, plastid divisions, retrograde signaling, etc. (Zhou et al., 2011). Photosynthesis and photosynthesis antenna proteins are important components of chloroplasts (Liu et al., 2020). A large number of proteins have also been found to be essential for chloroplast development, such as including light-harvesting chlorophyll and transcription factors (Waters and Langdale, 2009). In this study, the top three most highly enriched pathways were “Photosynthesis,” “Photosynthesis-antenna proteins,” and “Carbon fixation in photosynthetic organisms” (Figures 3A,B). All DEGs involved in photosystem and chlorophyll-binding proteins were highly expressed in the green leaf tissue. Ornamental kale exhibits a chlorophyll defect under low-temperature conditions. HSP70 is closely related to heat stress and is thought to mediate protein import in chloroplasts (Zhang and Glaser, 2002; Yue et al., 2021). There was a significantly higher expression level of *HSP70* in white leaf tissue, indicating that *HSP70* may be potential candidate genes related to temperature. GLK plays an important role in the regulation of chloroplast development (Xie et al., 2018). Genes of *GLK1* and *GLK2* were also highly expressed in the green leaf tissue. These results demonstrated that a disruption in chloroplasts development and function lead to white leaf formation, furthermore confirming previous reports.

The photosynthesis process relies on the cooperative interaction of two photosystems: photosystem I (PS I) and photosystem II (PS II). PS I and PS II contain several subunits, such as PSAE, PSAG, PSBQ, and PSBO, and these extrinsic proteins of PSII are known to be targets of stress (Sasi et al., 2018; Liu et al., 2021). PSBQ is responsible for multiple interactions with both PSII intrinsic and light-harvesting proteins (Sasi et al., 2018). In this study, higher *PSBQ* expression levels were observed in the green leaf tissue, indicating *PSBQ* may be an important factor affecting the green and white leaf formation of ornamental kale. Only a few studies have found *PSBQ* participated in leaf color regulation. Zhao et al. (2014) showed that *Bra040517* may be a candidate gene for *B. napus* chlorophyll-deficient mutant. PS I and PS II receive excitation energy from the light-harvesting complex (LHC) (Kohorn et al., 1986). The LHC antenna system of PS I and PS II is LHCA and LHCB, respectively. LHCs can greatly improve the efficiency of photosynthesis (Kargul and Barber, 2008). Our study showed that the genes encoding LHC were downregulated in the white leaf, especially *LHCB1.3* and *LHCB2.4*. Similar results were found by Zhou et al. (2011). These results indicated that the interference of photosynthesis results in leaves lacking green coloration.

The molecular mechanisms of white leaf formation are complex and related to multiple biochemical processes and genes in different ornamental kale cultivars. Yan et al. (2020) identified the cytochrome P450 gene (*Bol015404*) as the most likely candidate gene for white leaf formation of ornamental kale by using BSR-seq. In this study, this gene was a significantly high expression in white leaf tissue than that in the green leaf tissue [log_2_(W/G) = 12.071, log_2_(W1/G2) = 1.322]. These results are consistent but seem to be more closely with the comparison group of G02 vs. 1631. It is worth mentioning that in the study of Yan et al. (2020), a single peak occurred in chromosome C01, which may correspond to the *PSBQ* gene (*Bo1g158910*). This result further indicated that *PSBQ* may be involved in the formation of the white leaf.

## Conclusion

Summary, we performed transcriptomic analysis of ornamental kale cultivars with three different phenotypes *via* high-throughput Illumina sequencing. Results indicated that 24 chlorophyll metabolism and 15 carotenoid biosynthesis common DEGs were almost upregulated in green leaf tissue. In addition, green leaf tissue had higher levels of chlorophyll and carotenoids than white leaf tissue. Several genes related to chloroplast development and photosynthesis were expressed at high levels in green leaf tissue. Genes of *chlH*, *por*, *PSBQ*, *HSP70*, *LHCB1.3*, and *LHCB2.4* were potential candidate genes involved in leaf color formation. In conclusion, chlorophyll and carotenoid biosynthesis, chloroplast development and photosynthesis are the main metabolism process that affects the formation of green and white leaf tissue. These findings increase our understanding of the molecular mechanisms regulating green and white leaf pigmentation in ornamental kale.

## Data Availability Statement

The original contributions presented in the study are publicly available. This data can be found here: National Center for Biotechnology Information (NCBI) BioProject database under accession number, PRJNA761855.

## Author Contributions

PZ and XF designed the experimental trials. FZ and YL performed the experiments and collected the samples. YZ and FZ wrote the manuscript. All authors read and approved the final manuscript.

## Conflict of Interest

The authors declare that the research was conducted in the absence of any commercial or financial relationships that could be construed as a potential conflict of interest.

## Publisher’s Note

All claims expressed in this article are solely those of the authors and do not necessarily represent those of their affiliated organizations, or those of the publisher, the editors and the reviewers. Any product that may be evaluated in this article, or claim that may be made by its manufacturer, is not guaranteed or endorsed by the publisher.

## Footnotes

1
http://plants.ensembl.org/Brassica_oleracea/Info/Index
